# Smoking Status Modifies the Relationship between Th2 Biomarkers and Small Airway Obstruction in Asthma

**DOI:** 10.1155/2021/1918518

**Published:** 2021-11-28

**Authors:** Shuyuan Chu, Libing Ma, Jianghong Wei, Jiying Wang, Qing Xu, Meixi Chen, Ming Jiang, Miao Luo, Jingjie Wu, Lin Mai, Guofang Tang, Biwen Mo

**Affiliations:** ^1^Department of Respiratory and Critical Care Medicine, Affiliated Hospital of Guilin Medical University, Guilin, Guangxi, China; ^2^Laboratory of Respiratory Disease, Affiliated Hospital of Guilin Medical University, Guilin, Guangxi, China; ^3^Duke Global Health Institute, Duke University, Durham, NC, USA

## Abstract

**Background:**

Cigarette smoking and Th2-inflammation are both crucial in the pathogenesis of asthma. However, it is unknown whether smoking can affect the association between Th2-inflammation and small airway obstruction in adults with asthma.

**Methods:**

Adults diagnosed with asthma by a pulmonologist according to Global Initiative for Asthma guidelines were recruited from September 2016 to April 2018 to participate in this study. Participants were divided into two groups, the small airway obstruction group (those with FEF25–75% predicted value ≤ 65%) and the normal small airway function group (those with FEF25–75% predicted value > 65%). Final data analysis included 385 and 93 people in the Obstructive Group and the Normal Group, respectively. Total serum IgE level and blood eosinophil count were used as biomarkers of the Th2 phenotype.

**Results:**

The Obstructive Group had a larger fraction of smokers, higher blood eosinophil count, and lower lung function than the Normal Group. Current-smoking status was associated with an increased risk of small airway obstruction (adjusted odds ratio = 4.677, 95% confidence interval [1.593–13.730]); and log-IgE level was associated with a decreased risk of small airway obstruction (0.403 [0.216–0.754]). Smoking status stratified analysis showed an association between log-IgE level and a decreased risk of small airway obstruction only in never-smoker asthmatics (0.487 [0.249–0.954]).

**Conclusions:**

Current-smoking status and total serum IgE are, respectively, associated with small airway obstruction. Smoking status modifies the relationship between Th2 biomarkers and small airway function. These findings contribute to the understanding of risk factors associated with asthma endotyping.

## 1. Introduction

Asthma is a highly complex disease with unclear endotypes. It is known that Th2 response promotes development of asthma [1]. Eosinophil counts and IgE level in blood are widely accepted as reliable Th2 biomarkers in asthma diagnosis and management [2, 3]. Forced expiratory flow (FEF) at 25–75% predicted value (25–75%pred) has been used as an ancillary biomarker of small airway function in asthma management [2, 4]. FEF25–75%pred is related to asthmatic symptoms, bronchial hyper-reactivity, and blood eosinophilia [5]. Prebronchodilator FEF25–75%pred is also a sensitive indicator for the early detection, severity, and progression of asthma [6]. Interestingly, Th2-type cytokine gene polymorphisms are related with FEF25–75 value in asthma patients [7], suggesting that Th2 response may promote small airway obstruction. In this study, hence, we aim to investigate the relationship between Th2 biomarkers, namely, blood eosinophil counts and serum IgE level, and small airway obstruction based on FEF25–75%pred measurements.

Smoking is a well-established risk factor of asthma. The effect of smoking status on the burden of asthma has been increasingly recognized [8]. Although current smoking has been associated with increased mortality rate in asthma patients [9], we could not find studies that have investigated the role of smoking status (current smoking, ex-smoking, and never smoking) in the association between Th2-type biomarkers and small airway function in asthma patients. In this study, we also aim to examine whether smoking status modifies the relationship between Th2 inflammatory response and small airway function (FEF25–75%pred). We expect the findings of this study will help understand asthma endotypes involved in the pathways linking smoking, Th2-type inflammation, and small airway obstruction.

## 2. Methods

### 2.1. Participants Enrollment

We recruited participants in the Affiliated Hospital of Guilin Medical University, Guilin, China, from September 2016 to April 2018. All participants were adults (≥18 years old) who had been diagnosed with asthma according to the definition of Global Initiative for Asthma (GINA) guidelines [10]. Patients were excluded if they had chronic obstructive pulmonary disease (COPD), had a history of intubation within the prior 3 years, or had obstructive sleep apnea. The subjects were excluded as COPD patients when FEV1/FVC < 70% and had a reversibility of less than 15% after inhalation of 200 mg of salbutamol. Our study was approved by the Institutional Review Board (Ethics Committee) at the Affiliated Hospital of Guilin Medical University. Written informed consent was obtained from each participant.

Participants were divided into two groups based on their baseline FEF25–75%pred values. Those with a FEF25–75 ≤ 65%pred small airway obstruction (obstructive group) and group with normal small airway function (normal group) [11, 12].

In stratified analyses, subjects were classified into current smokers, never smokers, and ex-smokers. If the subjects had been in smoking cessation at least three months before they were recruited into our study, they were classified as ex-smokers in our study [13].

### 2.2. Assessment of Clinical Characteristics and Risk Factors

All subjects underwent standardized spirometry test (CareFusion™ MasterScreen Pneumo, Germany) according to the European Respiratory Society/American Thoracic Society standards [14]. FEF50, FEF50%pred, FEF75, FEF75%pred, FEF25–75, and FEF25–75%pred reflect small airway function. Forced expiratory volume in 1 second (FEV1), FEV1%pred, forced vital capacity (FVC), FVC%pred, and FEV1/FVC suggests airway obstruction. Peak expiratory flow (PEF) indicates upper airway resistance. The blood eosinophil count was assessed by Sysmex XN-2800^TM^ automated hematology analyzers (Sysmex America, Inc., USA). The total serum IgE was tested using Cobas e 801 analyzer with Elecsys IgE II (Roche Diagnostics, Germany) according to manufacturer's protocol. The cut-off value for high blood eosinophils was >400 uL and for high total IgE was >240 ng/ml. Health status was evaluated using the Asthma Quality of Life Questionnaire (AQLQ) [15] and the Short-Form 36 Questionnaire (SF-36) [16]. Asthma Control Test (ACT) [17] was used to assess symptom scores after the first-month initial treatment according to the GINA guideline.

### 2.3. Statistical Analysis

Group data were expressed as the mean ± standard deviation (SD) or median (range). Differences were evaluated using independent-samples *t* test or Mann–Whitney-U test for continuous variables, or chi square test for categorical variables. The association between blood eosinophil count, total serum IgE level, and small airway obstruction was assessed using unconditional logistic regression models with LOGISTIC procedure of SAS 9.4 (SAS Institute Inc., Cary, North Carolina, USA). The results were presented as odds ratios (OR) and 95% confidence intervals (CI). *P* values < 0.05 were considered statistically significant.

## 3. Results

A total of 478 subjects were selected for final analyses, when we excluded subjects if they had no records of FEF25–75%pred, blood eosinophil count, total serum IgE level, or smoking status (Supplementary Figure 1).

Table 1 illustrates baseline characteristics of subjects in the Obstructive Group and in the Normal Group. When compared with the Normal Group, the Obstructive Group subjects were older, had lower family income, were a higher fraction of ex and current smokers, and had higher blood eosinophil count, worse health status, and worse symptom score after the first-month initial treatment of asthma. In contrast, the Normal Group was showed a higher prevalence of inhaled corticosteroid (ICS) or long-acting beta-agonist (LABA) (ICS = 86, 92.5%; LABA = 86, 92.5%) than the Obstructive Group (ICS = 316, 82.1%; LABA = 315, 81.8%). In each group, there were three patients who took oral glucocorticoid methylprednisolone plus ICS. Moreover, the Obstructive Group had lower baseline lung function including small airway function than the Normal Group. As illustrated in Table 2, in the Obstructive Group, FEV1, FEV1%pred, FVC, FVC%pred, PEF, PEF%pred, FEF50, FEF50%pred, FEF75, FEF75%pred, FEF25–75, and FEF25–75%pred were all lower than the Normal Group.

We investigated the association between Th2-type biomarkers, smoking status, and small airway obstruction.  Table 3 shows that the status of current smoking was associated with an increased risk of small airway obstruction (adjusted OR = 4.677, 95% CI 1.593–13.730). Interestingly, log10 transformed IgE level in serum was associated with a decreased risk of small airway obstruction (adjusted OR = 0.403, 95% CI 0.216–0.754).

We further explored the association between Th2-type biomarkers and small airway function in stratified analysis by smoking status. In never smokers, we found an association between log10 transformed IgE level in serum and a decreased risk of small airway obstruction (OR = 0.487, 95% CI 0.249–0.954) (Table 4). This association was not found in smokers (ex or current smokers).

## 4. Discussion

In our study, the small airway obstructive group had a greater fraction of smokers, higher eosinophil count in blood, and lower lung function than the normal group. We found that current-smoking status was associated with an increased risk of small airway obstruction. Smoking history is related with abnormal peripheral airway function of adult patients with asthma [18]. Current smoking is associated with lower lung function of asthmatic patients [19, 20]. Furthermore, among adults with newly onset asthma, FEF25–75% is significantly reduced in current regular smokers and in recent (<1 year) ex-smokers when compared with never smokers [21]. This is due to pathological injury in the small airways by cigarette smoking. Cigarette smoking could promote small airway obstruction by enhancing mucin overproduction, lung inflammation, small airway epithelial-mesenchymal transition, and remodeling [22–24]. Our study is consistent with those previous findings and further demonstrates the association between current smoking and FEF25–75%pred, indicating an association between current smoking and an increased risk of small airway obstruction.

In this study, we also found that higher total serum IgE level was associated with a decreased risk of small airway obstruction. However, this IgE-airway obstruction association was only found in never smokers when stratified analyses were conducted by smoking status. Although the *P* value was 0.036 for that association, the 95% CI (0.216–0.754) further confirmed it and showed the strength of the effect. However, these results are not consistent with previous reports. Thus, it should be carefully deliberated. Previously total serum IgE level was found to be higher in smokers without asthma, whereas their FEF25–75%pred was lower than nonsmoking siblings [25]. Our study differs from this previous study, which included nonasthmatic subjects and did not separate ex-smokers or current smokers from all smokers. Furthermore, the authors assessed the effect among nonasthma subjects instead of asthma patients and did not investigate the effect of ex-smoking and current smoking. In contrast, a similar relationship was found in a previous study of 90 asthmatic subjects. It found that FEF25–75 value was higher in subjects with lower total serum IgE [26]. However, that study only analyzed subjects without smoking and current smoking, whereas our study included asthmatic subjects of never smoking, ex-smoking, or current smoking in a larger sample size. That may be related to the modification of smoking status in the association between IgE level and small airway obstruction. Thus, we further explored the effect of smoking status on the association between Th2 biomarkers and small airway obstruction by smoking status stratification.

When subjects were stratified on smoking status, our results showed an association between an increase of serum IgE level and a decreased risk of small airway obstruction in never-smoker asthmatic patients but not in current-smokers or ex-smokers. This finding suggests that IgE levels in current smokers or ex-smokers should be higher than that in never smokers in our study, perhaps partly due to the effect of smoking. It has been reported that smoking could attenuate the decrease of IgE level in steroid-naive patients with asthma [27]. In smoking-discordant monozygotic twins, total serum IgE is significantly higher in the smokers than in their nonsmoking siblings [25]. Studies using animal models of asthma found that cigarette smoke exposure could reduce allergen-induced total IgE in serum and Th2 response in the lung, in which nicotine played a key role [28, 29]. In contrast to total IgE in blood, blood eosinophil count is unchanged with cotinine exposure in asthma patients, even though blood eosinophil count is increased in controls [30]. Those may be the reasons that we observed the association between total serum IgE and small airway obstruction based on smoking status, but not blood eosinophil count. Therefore, our result suggests that smoking status may modify the association between IgE and small airway obstruction, which may be related with an endotype involved smoking, Th2-type inflammation, and small airway function. Thus, when total IgE level and small airway obstruction are assessed by pulmonologists, precise smoking status should be considered.

We acknowledge that we assessed eosinophil count and IgE level in blood, instead of eosinophils and Th2-type cytokines in the airway. Biomarkers in the airway could reflect local inflammation, whereas eosinophil count and IgE level in blood could indicate systematic inflammation. Since asthma is widely accepted as a systemic disease and systemic inflammation is a characteristic of asthma endotype, particularly in patients with serious symptoms [31], it may be more helpful to reflect asthma endotype that we assessed eosinophil count and IgE level in blood. Second, we did not assess Th2-type cytokines but only eosinophil count and IgE level in blood. Blood eosinophil count and total serum IgE level are classic biomarker of Th2 response, which could directly reflect phenotype of systematic inflammation.

## 5. Conclusions

Current-smoking status and total serum IgE are, respectively, associated with small airway obstruction. Smoking status modifies the relationship between Th2 biomarkers and small airway function. These findings contribute to further understanding of risk factors associated with asthma endotyping.

## Figures and Tables

**Table 1:** Baseline characteristics of subjects.

Variable	Total subjects (*n* = 478)	Obstructive group (*n* = 385)	Normal group (*n* = 93)	*P* value

Gender (male)	200 (41.8%)	165 (42.9%)	35 (37.6%)	0.360
Age (median[range])	45[18, 78]	46[18, 78]	41[18, 66]	0.003
BMI (kg/m2)	23.1 ± 3.3	23.1 ± 3.3	23.1 ± 3.2	0.840

Education (yrs)				
≤9	297 (62.1%)	239 (62.1%)	58 (62.4%)	0.299
10–12	70 (14.6%)	61 (15.8%)	9 (9.7%)	
13–16	102 (21.3%)	79 (20.5%)	23 (24.7%)	
≥17	9 (1.9%)	6 (1.6%)	3 (3.2%)	

Family income (10 thousand RMB/yr)				
<5.0	283 (59.2%)	236 (61.3%)	47 (50.5%)	0.019
5.0–9.9	101 (21.1%)	81 (21.0%)	20 (21.5%)	
10.0–19.9	75 (15.7%)	51 (13.2%)	24 (25.8%)	
≥20.0	12 (2.5%)	11 (2.9%)	1 (1.1%)	
Smoking history (yes)	129 (27.0%)	114 (29.6%)	15 (16.1%)	0.001
Smoking history (pack-year)	6.3 ± 14.0	7.1 ± 14.8	2.9 ± 9.3	0.001

Smoking status				
Current smoking	79 (16.5%)	70 (18.2%)	9 (9.7%)	0.031
Ex-smoking	50 (10.5%)	44 (11.4%)	6 (6.5%)	
Never smoking	349 (73.0%)	271 (70.4%)	78 (83.9%)	
Blood eosinophil count (uL)	325 ± 405	345 ± 435	242 ± 230	0.028
Blood eosinophils >400 uL	132 (27.6%)	115 (29.9%)	17 (18.3%)	0.025
Total IgE in blood (ng/ml)	775.805 ± 898.192	742.625 ± 870.746	913.163 ± 997.238	0.100
Total IgE >240 ng/ml	354 (74.1%)	279 (72.5%)	75 (80.6%)	0.106
Blood eosinophils >400 uL plus total IgE >240 ng/ml	106 (22.2%)	91 (23.6%)	15 (16.1%)	0.118

ICS (yes)	402 (84.1%)	316 (82.1%)	86 (92.5%)	0.014
LABA (yes)	401 (83.9%)	315 (81.8%)	86 (92.5%)	0.012
LTRA (yes)	25 (5.2%)	18 (4.7%)	7 (7.5%)	0.396

AQLQ				
Symptoms score	5.1 ± 1.1	5.0 ± 1.1	5.6 ± 1.0	<0.001
Activity limitation score	5.2 ± 1.1	5.2 ± 1.1	5.6 ± 1.0	<0.001
Emotional function score	5.3 ± 1.2	5.2 ± 1.2	5.6 ± 1.1	0.002
Environmental stimuli score	4.8 ± 1.3	4.7 ± 1.3	5.0 ± 1.3	0.084
Total	5.2 ± 1.0	5.1 ± 1.0	5.5 ± 0.9	<0.001

SF-36				
Bodily pain	1.9 ± 1.1	2.0 ± 1.1	1.8 ± 1.0	0.102
Physical functioning	26.3 ± 3.6	26.0 ± 3.7	27.4 ± 3.0	<0.001
Physical role	6.8 ± 1.8	6.6 ± 1.8	7.3 ± 1.4	<0.001
General health	15.7 ± 1.4	15.7 ± 1.4	15.4 ± 1.2	0.117
Vitality	15.4 ± 2.3	15.4 ± 2.2	15.3 ± 2.4	0.716
Social functioning	7.0 ± 1.2	7.0 ± 1.2	7.0 ± 1.4	0.901
Emotional role	5.2 ± 1.3	5.1 ± 1.3	5.6 ± 1.0	0.001
Mental health	20.0 ± 2.5	19.9 ± 2.5	20.3 ± 2.5	0.260
Reported health transition	3.3 ± 0.9	3.4 ± 0.9	3.1 ± 1.0	0.008
Total	101.5 ± 7.3	101.1 ± 7.4	103.1 ± 6.6	0.015
ACT score^*∗*^	18.7 ± 3.9	18.4 ± 3.9	20.2 ± 3.5	<0.001

^*∗*^ACT score after the first-month initial treatment. BMI, body mass index. ICS, inhaled glucocorticoid. LABA, long-acting beta-agonist. LTRA, leukotriene receptor antagonists. AQLQ, Asthma Quality of Life Questionnaire. SF-36, Short-Form 36 Questionnaire. ACT, asthma control test.

**Table 2:** Baseline lung function of subjects.

Variable	Total subjects (*n* = 478)	Obstructive group (*n* = 385)	Normal group (*n* = 93)	*P* value

Preinhaling short-acting bronchodilator				
FVC (L)	2.91 ± 0.84	2.80 ± 0.80	3.41 ± 0.83	<0.001
FEV1 (L)	2.00 ± 0.75	1.82 ± 0.66	2.77 ± 0.63	<0.001
FEV1%pred (%)	69.48 ± 22.40	63.20 ± 18.59	95.48 ± 17.65	<0.001
FEV1/FVC (%)	67.48 ± 12.86	64.03 ± 11.50	81.80 ± 6.95	<0.001
PEF (L/s)	4.56 ± 1.92	4.15 ± 1.78	6.25 ± 1.54	<0.001
PEF%pred (%)	57.64 ± 21.75	51.97 ± 18.47	81.23 ± 18.29	<0.001
FEF50 (L/s)	1.79 ± 1.12	1.42 ± 0.82	3.30 ± 0.92	<0.001
FEF50%pred (%)	43.10 ± 24.75	34.40 ± 16.80	79.04 ± 19.30	<0.001
FEF75 (L/s)	0.66 ± 0.49	0.51 ± 0.35	1.28 ± 0.50	<0.001
FEF75%pred (%)	37.18 ± 23.32	28.62 ± 14.24	72.15 ± 20.38	<0.001
FEF25–75	1.50 ± 0.98	1.18 ± 0.70	2.84 ± 0.80	<0.001
FEF25–75%pred (%)	41.76 ± 24.73	31.91 ± 14.58	82.53 ± 14.05	<0.001

Postinhaling short-acting bronchodilator				
FVC (L)	3.09 ± 0.87	2.94 ± 0.85	3.70 ± 0.70	<0.001
FEV1 (L)	2.40 ± 0.91	2.18 ± 0.82	3.32 ± 0.64	<0.001
FEV1%pred (%)	84.42 ± 30.79	76.51 ± 26.02	117.16 ± 27.25	<0.001
FEV1/FVC (%)	73.97 ± 17.35	70.05 ± 16.25	90.19 ± 11.31	<0.001
PEF (L/s)	4.80 ± 1.90	4.42 ± 1.78	6.39 ± 1.50	<0.001
PEF%pred (%)	60.97 ± 22.25	55.46 ± 19.05	83.92 ± 19.84	<0.001
FEF50 (L/s)	2.02 ± 1.15	1.66 ± 0.87	3.50 ± 0.99	<0.001
FEF50%pred (%)	49.07 ± 25.91	40.43 ± 18.11	84.72 ± 22.59	<0.001
FEF75 (L/s)	0.74 ± 0.50	0.59 ± 0.37	1.38 ± 0.49	<0.001
FEF75%pred (%)	42.94 ± 25.21	33.92 ± 16.44	79.78 ± 20.96	<0.001
FEF25–75%	1.66 ± 0.99	1.33 ± 0.70	3.01 ± 0.83	<0.001
FEF25–75%pred (%)	46.75 ± 26.48	36.67 ± 16.29	88.48 ± 18.56	<0.001

FEV1, forced expiratory volume in 1 second. FVC, forced vital capacity. PEF, peak expiratory flow. FEF, forced expiratory flow. %pred, % predicted.

**Table 3:** Association between Th2 biomarkers, smoking status, and small airway obstruction.

Variables	Unadjusted			Adjusted		
	OR	95% CI	*P* value	OR	95% CI	*P* value

Current smoking^a^^*∗*^	2.239	1.070–4.684	0.032	4.677	1.593–13.730	**0.005**
Ex-smoking^b^^*∗*^	2.111	0.867–5.137	0.100	2.316	0.760–7.055	0.140
Lg (Eos)^c^	1.560	1.057–2.303	0.025	1.520	0.948–2.437	0.082
Lg (IgE)^d^	0.636	0.392–1.032	0.067	0.403	0.216–0.754	**0.004**

Exposure category: adjusted for sex, age, BMI, family income, education level, and asthma grade, including ^a^Lg (Eos), Lg (IgE); ^b^Lg (Eos), Lg (IgE); ^c^smoking status, Lg (IgE); ^d^smoking status, Lg (Eos). ^*∗*^Reference is never-smoking status. Lg (IgE), log10 transformed total IgE level in serum. Lg (Eos), log10 transformed eosinophil count in blood. Bold shows statistically significant value.

**Table 4:** Association between Th2 biomarkers and small airway obstruction by smoking status.

Variables	Unadjusted			Adjusted		
	OR	95% CI	*P* value	OR	95% CI	*P* value

Current smoking						
Lg (Eos)^a^	1.929	0.564–6.603	0.295	0.473	0.062–3.616	0.471
Lg (IgE)^b^	0.558	0.131–2.386	0.432	0.113	0.009–1.358	0.086

Ex-smoking						
Lg (Eos)^a^	1.275	0.302–5.386	0.741	1.422	0.259–7.791	0.685
Lg (IgE)^b^	0.266	0.028–2.562	0.252	0.065	0.002–2.706	0.151

Never smoking						
Lg (Eos)^a^	1.588	1.029–2.452	0.037	1.612	0.953–2.725	0.075
Lg (IgE)^b^	0.683	0.399–1.168	0.164	0.487	0.249–0.954	**0.036**

Exposure category: adjusted for sex, age, BMI, family income, education level, and asthma grade, including ^a^Lg (IgE); ^b^Lg (Eos). Lg (IgE), log10 transformed total IgE level in serum. Lg (Eos), log10 transformed eosinophil count in blood. Bold shows statistically significant value.

## Data Availability

The dataset generated and/or analyzed during the current study are not publicly available but are available from the corresponding author on reasonable request.

## Supplementary Materials

Supplementary Materials
*Supplementary Figure 1*. Selection of study subjects. FEF, forced expiratory flow. %pred, % predicted. STROBE Statement, checklist of items that should be included in reports of cohort studies.
