# Supplementary figures and images for: Smoking Status Modifies the Relationship between Th2 Biomarkers and Small Airway Obstruction in Asthma
Source: Can Respir J. 2021 Nov 28;2021:1918518. doi: 10.1155/2021/1918518 (PMC8645388; doi:10.1155/2021/1918518)

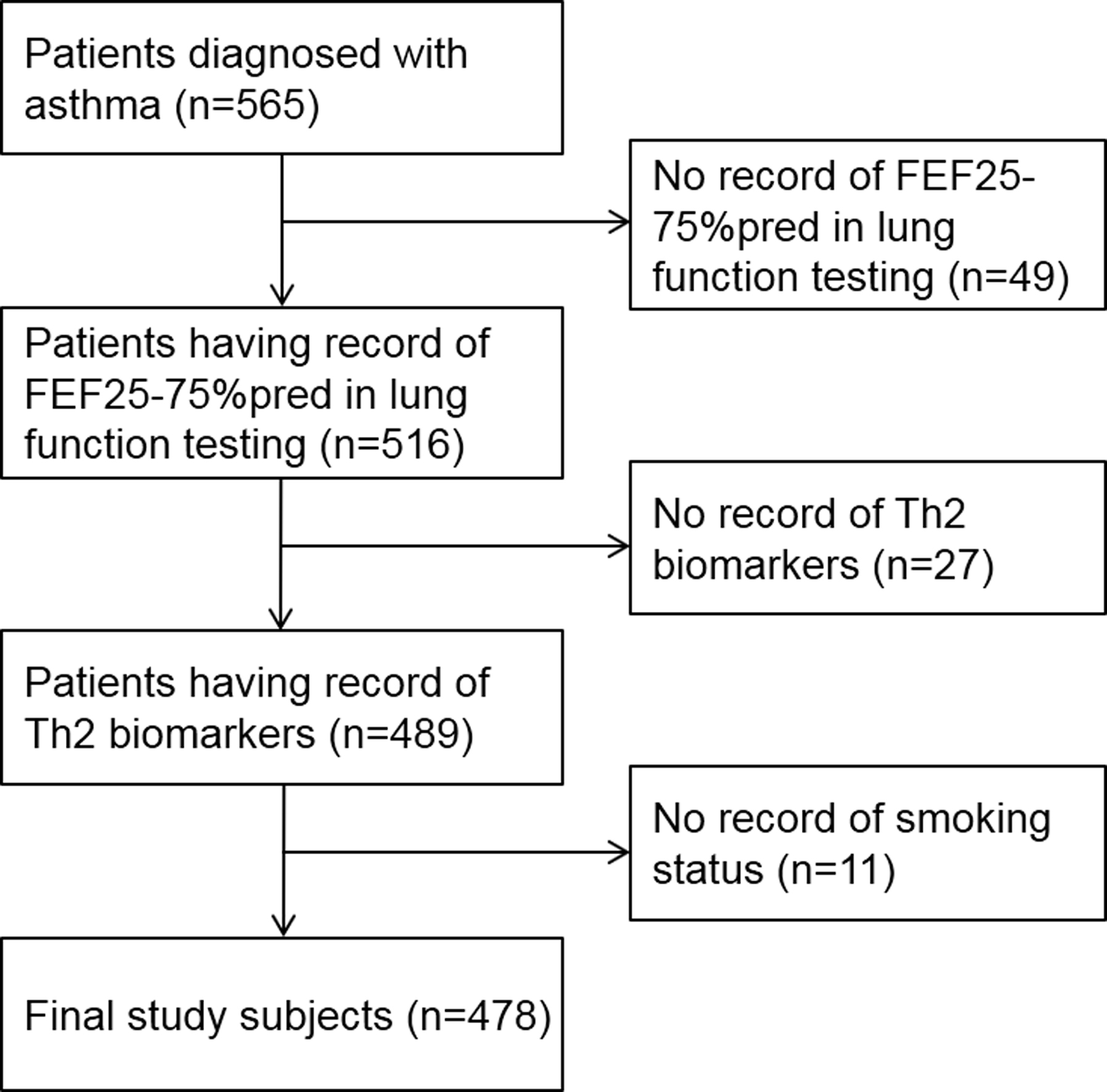

Supplement: Supplementary Materials — Supplementary Figure 1. Selection of study subjects. FEF, forced expiratory flow. %pred, % predicted. STROBE Statement, checklist of items that should be included in reports of cohort studies. [file 1918518.f1.zip › 1918518.f1/figure 1.jpg]
